# Dual n-back training improves functional connectivity of the right inferior frontal gyrus at rest

**DOI:** 10.1038/s41598-020-77310-9

**Published:** 2020-11-23

**Authors:** Tiina Salminen, Caroline Garcia Forlim, Torsten Schubert, Simone Kühn

**Affiliations:** 1 Department of Psychology, Faculty of Life Sciences, Humboldt-Universität zu Berlin, Rudower Chaussee 18, 12489 Berlin, Germany; 2 Clinic and Policlinic for Psychiatry and Psychotherapy, University Clinic Hamburg-Eppendorf, Martinistraße 52, 20246 Hamburg, Germany; 3 Department for Psychology, Martin-Luther University Halle-Wittenberg, Emil-Abderhalden Str. 26, 06108 Halle (Saale), Germany; 4 Lise Meitner Group for Environmental Neuroscience, Max Planck Institute for Human Development, Lentzeallee 94, 14195 Berlin, Germany

**Keywords:** Cognitive neuroscience, Working memory, Human behaviour

## Abstract

Several studies have shown that the benefits of working memory (WM) training can be attributed to functional and structural neural changes in the underlying neural substrate. In the current study, we investigated whether the functional connectivity of the brain at rest in the default mode network (DMN) changes with WM training. We varied the complexity of the training intervention so, that half of the participants attended dual n-back training whereas the other half attended single n-back training. This way we could assess the effects of different training task parameters on possible connectivity changes. After 16 training sessions, the dual n-back training group showed improved performance accompanied by increased functional connectivity of the ventral DMN in the right inferior frontal gyrus, which correlated with improvements in WM. We also observed decreased functional connectivity in the left superior parietal cortex in this group. The single n-back training group did not show significant training-related changes. These results show that a demanding short-term WM training intervention can alter the default state of the brain.

## Introduction

The benefits of working memory (WM) training have been established in numerous studies, showing that WM training interventions may not only improve WM performance but also task-unrelated cognitive functions^1–4^. The behavioral improvements are often accompanied by changes of the underlying neural substrate, such that both functional and structural changes can be observed and attributed to the training intervention. More specifically, such training has been shown to alter functional activation in different brain regions and task-related connectivity between regions^5–8^. Furthermore, WM training has been shown to increase white matter integrity in several white matter connections, providing evidence for increased structural connectivity between different brain regions^9,10^.

These neural findings have provided strong evidence that the benefits of WM training can be attributed to the plasticity of the underlying neural substrate. However, not only task-related activation changes reflect the mechanisms of training effects, but also the changes in neural functions while the brain is at rest (i.e. not performing a task) may provide important information on how the brain reorganizes itself through experience. Resting state functional connectivity predicts performance in cognitive tasks^11^ and therefore it can be assumed to change in response to cognitive training interventions.

To retrieve the resting state networks, a most reliable method is the independent component analysis (ICA)^12,13^. ICA possesses important advantages in comparison to other methods as seed functional connectivity: it does not depend on prior assumptions as the choice of seed region or a predefined template to account for the brain regions. ICA is, therefore, a voxel-wise data-driven method. In addition, signals not related to brain activity are removed, as movement, blinking, heartbeat and breathing artefacts.

These advantages come to the fact that ICA blindly decomposes brain signals into multiple independent components. The central idea is to separate signals coming from different sources of activity into independent components. Each component, or source of activity, has a temporal part with an associated spatial map. The spatial map is a grouping of voxels with synchronized activity, representing a temporally coherent functional network^12,13^. According to this spatial grouping of voxels, the components are associated with sources that can be related either to brain activity or to noise. The components associated with brain activity-related sources resemble discrete cortical functional networks and are named resting state networks.

Previous studies using ICA on resting state data have identified several resting state networks consisting of different brain regions that share similar activity patterns while the participant is not performing any task^14,15^. In these studies, the use of ICA in combination with more or less predefined assumptions about the network’s structural components allows for disentangling the contribution of potential constituting parts of the neural networks. Most of these networks have been associated with different functions, such as the sensory/motor network, the executive control network, and the visual and auditory networks.

Whereas these resting-state networks can be identified while the brain is at rest, the default mode network (DMN) is the only network that shows decreased activity during task performance^16^. The DMN are formed by key cortical regions such as medial prefrontal cortex, inferior parietal cortex, the posterior cingulate cortex, precuneus, hippocampus, insula. Due to its activation at rest and deactivation during tasks, the DMN can be described as reflecting a default state of the brain.

Resting-state brain function is sensitive to developmental as well as pathological changes in the brain^17^. However, only a few studies have investigated the effects of cognitive training interventions on the intrinsic functional architecture of the brain. Takeuchi et al.^18^ showed increased resting-state activation in the medial prefrontal cortex (mPFC) and precuneus nodes of the DMN after a 4-week WM training intervention. The changes were, however, compared against a passive control group (i.e., no-training group), which leaves open, to what degree the observed changes can be attributed to WM training per se, and not for example to the sole activity of taking part in an intervention (however, see^19^ for similar effects in children). Furthermore, the training intervention in the study was very demanding, requiring the engagement of several cognitive functions (WM, executive functions, divided attention), and therefore it is difficult to disentangle, to which characteristics of the intervention the changes in resting-state activations can be attributed.

In the current study, we examined changes in the spontaneous neural activations in resting-state networks following different types of WM training interventions, and compared the effects between the different training groups as well as between the training groups and a passive control group. This approach enables to disentangle intervention effects from specific effects of training characteristics.

Two groups of participants attended a WM training intervention. The first group practiced on the dual n-back task, which in previous studies has been shown to train WM and to improve also other cognitive functions as well as to alter neural functions and brain structure^1,6,7^. The other group was trained on single n-back tasks, which consisted of the component task of the dual n-back but did not include the dual-task and divided attention components and, therefore, represents an appropriate active control for the training of cognitive control demands. In other words, we consider the dual n-back task to be more demanding to the cognitive system due to its additional cognitive components as compared to the single n-back tasks. A third group, a passive control group, attended only the pre- and post-assessments without taking part in any intervention between the sessions. The resting-state activity of the participants was assessed using fMRI measure before and after the training interventions. In order to assess the effects of possible changes in resting connectivity related to task-independent WM processes, participants performed in pre- and post-sessions a WM transfer task that also engages WM but differs from the training task with respect to the stimuli and cognitive processing components (see “Materials and methods”).

We hypothesized that WM training interventions would produce changes in resting-state activity, as compared with the effect of attending no intervention. Additionally, we expected that the changes in functional connectivity at rest could be differentiated between the two different training interventions. In particular, due to the executive control components of the training tasks, we expected a training-related connectivity increase in prefrontal regions. We further hypothesized that the increased post-training prefrontal resting connectivity in the dual n-back training group would exceed that of the single n-back training group, since the dual n-back task engages additional executive functions, such as divided attention and dual-task demands that are not present in the single n-back tasks.

This hypothesis was based on findings that have shown an important role of prefrontal brain regions in executive control functions and working memory^20–22^. Additionally, recent studies have provided evidence that prefrontal regions and their connectivity to other brain regions may undergo substantial plasticity under certain conditions, e.g. in the context of physical exercise^23^ and visual perceptual learning^24^.

## Materials and methods

### Subjects

The study protocol and procedures were reviewed and approved by the institutional review committee (Ethics Committee of the Department of Psychology). The procedures followed were in accordance with the ethical standards of the responsible committee on human experimentation (institutional and national) and with the Helsinki Declaration of 1975, as revised in 2008. All participants provided written informed consent.

We investigated 54 participants, who were randomly assigned to three different training intervention conditions. Eighteen participants took part in the dual *n*-back training condition (mean age 24.4 years, SD 4.0 years, age range 20–32 years, six male). As we were specifically interested in the significance of the unique complexity of the task due to the dual-task component, another 18 participants were assigned to a single *n*-back training condition (mean age 24.1 years, SD 3.1 years, age range 19–29 years, four male), and this group trained on the single subtasks of the dual *n*-back—that is, the auditory-verbal (AV) and the visuospatial (VS) *n*-back tasks—separately. As a third group, 18 participants (mean age 25.0 years, SD 4.0 years, age range 19–33 years, seven male) were assigned to a passive (no-contact) control group that did not undergo any training but attended only the pre- and posttest sessions. The groups did not differ significantly in age or gender distribution (both *p*’s > 0.54). All participants were right-handed and reported normal or corrected-to-normal vision and normal hearing and received a compensation of 8 €/h for their participation.

### Procedure

The training group trained on the dual *n*-back task each day for 30 min and the active control group trained on each of their training task (the AV and the VS single *n*-back tasks) each day for 30 min. As the training time of the active control group was thus altogether approximately 60 min each day, the training group watched a 30-min documentary film at the end of each training session. This was due to two reasons: in order to have the dual n-back training group spend as much time in the lab environment as the single n-back training group, but also to ensure that they are confronted with a cognitive task for as long a period as the single n-back training. We instructed the dual n-back trainees that there may be questions posed about the videos at a later point, in order to encourage the participants to truly focus on the content of the videos.

Previous literature has shown significant training and transfer effects after 3 weeks or 8 h of training^1,3^. Therefore, the training dose in the present study adds up to altogether 8 h, and thus is in accordance with the recommendations of these studies.

Before the first experimental session, each participant was informed about the experimental procedure. Additionally, the participants filled out a questionnaire to ensure their suitability for MRI-measurement, they were informed about the MRI-method and they signed an informed consent form. At the end of the first session the participants were explained the instructions for the fMRI tasks and finally they practiced each task for two runs. The participants in the training group and the active control group were at this point not yet informed about what their training task would be.

Before and after the training period all participants attended an MRI-scanning session. Post-training scanning session took place on the day directly following the final training day (equivalently for the passive control group who did not attend training).

### Training tasks

#### Dual *n*-back task

The dual *n*-back consisted of simultaneously presented auditory-verbal (AV) and visuo-spatial (VS) *n*-back tasks^1^. In the AV task, participants were presented with phonemes through headphones, and in the VS task, they saw blue squares in eight different locations on the computer screen. The phonemes and squares were presented simultaneously so that each stimulus appeared for 500 ms followed by a 2500 ms inter-stimulus interval (ISI).

Each training session started on the *n*-back level 2 so that the participants were instructed to react whenever a currently presented item was the same as the item presented two steps back. However, in each training session the task was adaptive, so that when the participant responded at least 90% correct in both tasks, they advanced to the next level (e.g. from 2-back to 3-back). If the participant responded 70% or less correct during a run in either of the tasks, they fell to a lower level (e.g. from 3-back to 2-back), the lowest possible level being 1-back. In any other case, the *n*-back level remained constant.

Each training session comprised 20 runs, and each run included 20 + *n* trials (e.g. a 2-back run consisted of 22 trials). Participants were instructed to press the key “L” with the right index finger for the AV targets and the key “A” with their left index finger for the VS targets. The task was self-paced and participants could start a new block by pressing the spacebar.

Participants received feedback on their performance after each block and they were informed about the *n*-back level of the next run.

#### Single *n*-back tasks

The single *n*-back tasks were the component (AV and VS) tasks of the dual *n*-back task. The stimuli, response keys and mappings, starting *n*-back level, feedback, and rules of adaptiveness were equal to the dual *n*-back task. However, the active control group never practiced the AV and VS task simultaneously, but in each training session, the tasks were trained as single tasks with 20 runs of each task. The order of the AV and the VS task was counterbalanced so that every other training session started with one of the tasks and every other session with the other task.

### fMRI tasks

In the scanner as the participants lay on their backs, the two response devices (one for each hand) were placed on their legs. Both devices included four response buttons that were placed horizontally next to each other. In the *n*-back task only the innermost buttons were needed, that is, the innermost button of the left response device for the left forefinger, and the innermost button of the right response device for the right forefinger. In the WM transfer tasks, all buttons were required, so that there was one button for each finger except for the thumbs.

#### *n*-Back tasks

The stimuli in the fMRI *n*-back tasks were the same as in the training tasks. However, the tasks were not adaptive and the *n*-back levels were 0- and 2-back in all versions of the *n*-back task: single AV, single VS, and the dual *n*-back. In the 0-back task, the participants were instructed to respond to a certain letter (AV) and to a certain position of the blue square (VS). There were six blocks à ten *n*-back runs of each task (an *n*-back run refers to one condition in the task, e.g. VS 2-back), and each run included ten trials. Thus, there were altogether 18 task blocks. The blocks were presented in random order with a task instruction screen presented before each block. Responses were given for targets only using the right index finger for the AV stimuli and the left index finger for the VS stimuli (in accordance with the training task).

#### WM transfer tasks

The WM transfer task was based on the letter memory task by Miyake et al.^25^. It consisted of three blocks: an AV, a VS and a dual-modality block in which the AV and VS stimuli were presented simultaneously. The order of the AV and the VS blocks was counterbalanced, half of the participants started with the AV block and the other with the VS block; all participants performed the dual-modality block as last.

In the AV task, the participants were presented with the numbers 1, 2, 3, and 4 through headphones sequentially in a random order. After each sequence, the participants were asked to report the last four numbers in the correct order, starting from the fourth last and ending with the last presented item. The participants were asked to respond as correctly as possible, however keeping in mind that after eight seconds from the question to report the last four items the task would continue automatically. Participants used their right hand to give responses: index finger for one, middle finger for two, ring finger for three and little finger for four. After each response phase a fixation cross was presented for 10 s and this time was used as a baseline period in the fMRI analyses.

The procedure of the VS block was similar to the AV block, but the stimuli consisted of black bars presented one by one in four different locations from bottom to top on the screen. The participants used their left hand for the responses: the index finger for a bar presented in the lowermost part of the screen, the middle finger for a bar presented slightly below the midline of the screen, the ring finger for a bar presented slightly above the screen, and the little finger for a bar presented on the uppermost part of the screen.

For each participant, the order of the AV and the VS block was the same at pre- and at posttest. All blocks included nine sequences of items, and the lengths of the sequences varied randomly so that a sequence could comprise 7, 9, 11, 13, or 15 items. The participants were unaware of the length of the ongoing sequence and they were asked to update constantly the contents of their WM.

Within the present study, we only used the behavioral data from the fMRI tasks (training n-back task and WM transfer task). The fMRI data has been reported separately^6^.

### fMRI data acquisition

Images were acquired with a 3.0 T Siemens Magnetom Trio–scanner using a 12-channel radiofrequency head coil. First, high-resolution T1-weighted 3D MPRAGE structural volumes were collected (repetition time = 2500 ms, echo time = 4.77 ms, acquisition matrix = 256 × 256 × 176, flip angle = 7°, voxel size = 1 × 1 × 1 mm^3^). Resting state data was acquired after the T1 image and before fMRI task activity were acquired. We acquired whole brain functional images while participants were asked to keep their eyes closed and relax for 5 min. We used a T2*-weighted echo-planar imaging (EPI) sequence (repetition time = 2000 ms, echo time = 30 ms, image matrix = 64 × 64, field of view = 216 mm, flip angle = 80°, slice thickness = 3.0 mm, distance factor = 20%, voxel size = 3 × 3 × 3 mm^3^, 36 axial slices, using GRAPPA). Images were aligned to the anterior–posterior commissure line.

### Resting state data analyses

#### Preprocessing

To ensure for steady-state longitudinal magnetization, the first 10 images were discarded. The acquired data was corrected for slice timing and realigned. Structural images were coregistered to functional images and segmented. Data was then spatially normalized to Montreal Neurological Institute (MNI) space and spatially smoothed with a 6-mm FWHM to improve signal-to-noise ratio. Movement and signal from white matter and cerebrospinal fluid were regressed. To reduce physiological high-frequency respiratory and cardiac noise and low-frequency drift data was filtered (0.01–0.09 Hz) and, finally, detrended. All steps of data preprocessing were done using SPM12 (Wellcome Department of Cognitive Neurology) except filtering that was applied using REST toolbox^26^. In addition, to control for motion, the voxel-specific mean framewise displacement (FD) was calculated according to Power and colleagues^27^. FD values were below the default threshold of 0.5 for all groups (Group dual training: before training = 0.109 ± 0.044 and after training = 0.106 ± 0.058, group single training: before training = 0.108 ± 0.040 and after training = 0.108 ± 0.051, group no training: first time point = 0.111 ± 0.050 and second time point = 0.108 ± 0.067).

#### Independent component analysis

ICA is a reliable method of extracting resting state networks of the brain. Each component (source) that is retrieved by ICA is a spatial grouping of voxels with synchronized activity, representing a temporally coherent functional network^12,13^. According to this spatial grouping of voxels, the components are associated with sources that can be related to either brain activity, as the DMN or noise.

ICA was performed in GIFT software (https://icatb.sourceforge.net/;^28^) using Infomax algorithm. For the purpose of ICA, preprocessed data from all individuals were used. The number of spatially independent resting-state networks (N) was estimated by the GIFT software (N = 21). The identification of the components retrieved by ICA was first done automatically by the GIFT toolbox, correlating each component’s spatial map with predefined RSN’s spatial map templates from the toolbox and then by experts (CGF and SK). In this study we focused on the spatial map of the DMN.

The spatial map of the DMN (comprising key cortical regions such as the posterior cingulate cortex, precuneus, medial prefrontal cortex, hippocampus, insula, and inferior parietal cortices), can be divided into ventral and dorsal DMN, depending on whether the frontal areas or the parietal areas were more prominent. In our study, the spatial map of the ventral DMN had higher correlation (R = 0.4) with the predefined spatial template provided by the GIFT toolbox.

For the group comparison, spatial maps were taken to the second level analysis in SPM12 (factorial design 3 groups (dual-task, single-task, control) with 2 levels (before and after training), *p* = 0.001 uncorrected and, additionally, thresholded with *p* ≤ 0.01 cluster level FWE, extent cluster size > 10, additional analysis using partitioned error model in the supplementary material, table S1). Mean FD, age, and sex were used as covariates in the second level analysis.

We were interested in two particular contrasts: (1) effect of dual-task training: the interaction of group × time comparing the dual-task training group with the single-task training group, for which we predicted increases selectively in the dual-task group, (2) effect of training in general: the interaction of group × time comparing the dual- and the single-task training group jointly against the passive control group, for which we predicted increases selectively in the two training groups. Using a family wise error (FWE) threshold on the cluster level of *p* < 0.01 we ran the two contrasts in both the positive and the negative direction on the spatial maps.

In order the investigate a possible correlation between functional connectivity calculated using ICA and the behavioral performance, we extracted the ICA values from the resulting cluster on the spatial maps showing a significant time × group interaction and correlated them to the behavioral performance scores derived from the n-back and working memory updating task performed during fMRI acquisition. The ICA values, are here defined as the values extracted from the spatial maps of the DMN. The correlation was calculated using non-parametric Spearman correlation coefficients.

We restricted the analysis to behavioral scores in which we found training effects in the single-task and/or the dual-task group, namely in dual n-back, auditory single n-back, visual single n-back and dual WM transfer updating task (see^6^ for details on the behavioral data). More precisely, we correlated the training-related gain in ICA extracted connectivity values of the spatial maps with the training-related gain in behavioral performance separately for each group.

## Results

### Behavioral results

Due to a technical failure, the data of one participant from the training group was not recorded in the pretest session of the dual *n*-back task, and the data of one participant from the passive control group was not recorded in the pretest session of the WM updating tasks. We excluded the data of these participants from the analyses of the corresponding tasks.

First, a multivariate analysis of variance (MANOVA, Pillai’s Trace) was conducted, with Group (training vs. active control vs. passive control) as a between*-*subjects factor and Session (pretest vs. posttest) as a within*-*subjects factor on the data of all tasks as dependent variables (i.e. the mean level of *n* in the behavioral *n-*back tasks and the number of correctly reported items in the WM updating tasks). This analysis revealed significant main effects of Session [*F*(6, 44) = 56.20, *p* < 0.001, η^2^_p_ = 0.89] and Group [*F*(12, 90) = 2.11, *p* < 0.05, η^2^_p_ = 0.22]. Importantly, the Group × Session interaction was significant [*F*(12, 90) = 6.45, *p* < 0.001, η^2^_p_ = 0.46], which indicated that there were reliable group-specific performance changes from pre- to posttest.

**Figure 1:**
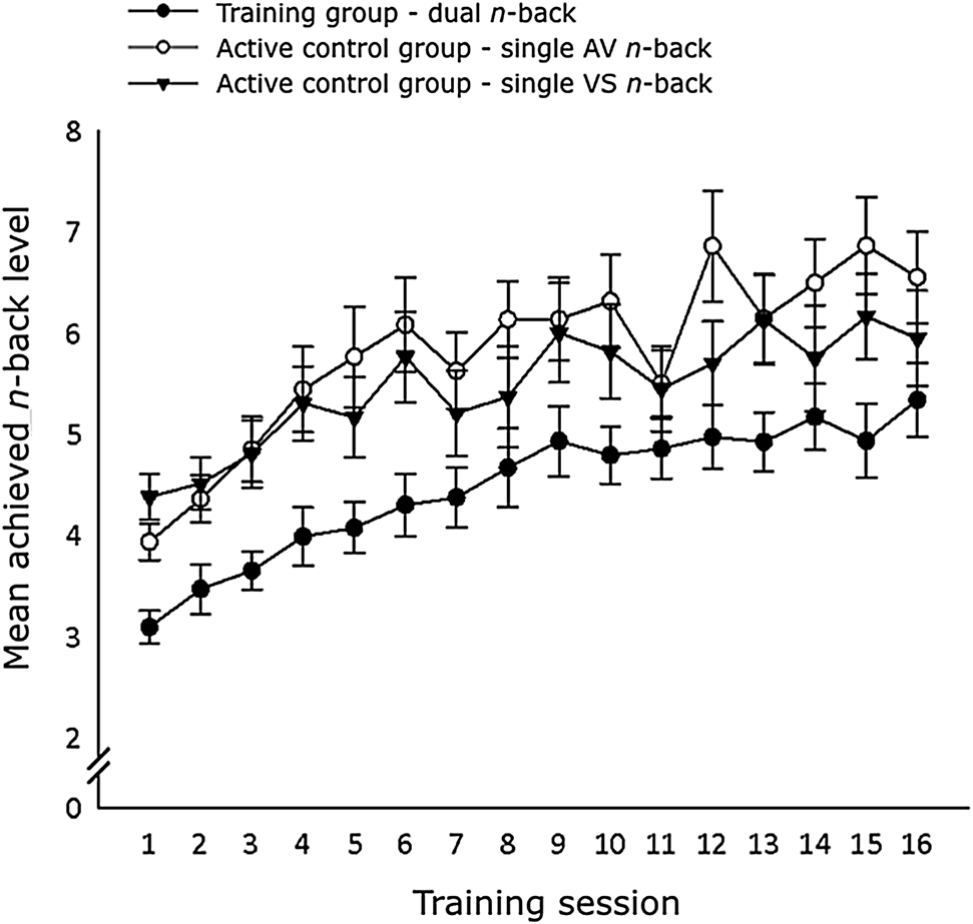
Improvement of the training group in the dual n-back task and of the active control group in the single tasks (AV and VS). For each training session, the mean achieved n-back level is depicted. Error bars indicate standard error of the mean.

A separate analysis for the training data showed that both the training and the active control group improved in their respective training tasks, as revealed by a significant main effect of Session [F(1, 19) = 68.66, *p* < 0.001, η^2^_p_ = 0.78 in the training group and F(1, 17) = 38.60, *p* < 0.001, η^2^_p_ = 0.69 in the active control group], Fig. 1. Note that the generally better performance of the active control group in their training tasks (i.e., the single subtasks) as compared to the training group’s performance in the dual n-back task likely reflects larger cognitive demands in the dual n-back task than in the single n-back tasks.

An analysis in the transfer tasks on the number of correctly reported 4-item sequences in each task (AV, VS, and dual) revealed that while in the single WM updating tasks all groups improved their performances equally from pre- to post-test (main effect of Session: F(1, 50) = 26.01, *p* < 0.001, η^2^_p_ = 0.28 for the AV task and *F*(1, 50) = 10.77, *p* < 0.01, η^2^_p_ = 0.18 for the VS task, Session × Group interaction: n.s. for both tasks), in the dual WM updating task only the training group showed a significant improvement [*t*(17) =  − 4.68, *p* < 0.001, Cohen’s *d* = 1.03] and there were no performance changes between the sessions in the active or the passive control groups (both *p*’s > 0.39).

The behavioral results thus show that the training group’s improvement in the trained dual n-back task transferred to an untrained dual WM task; training on the single n-back tasks did not produce such a transfer effect. These findings are suggestive for related changes in the connectivity (i.e., resting state) data. For a more comprehensive report on the behavioral analyses, please see^6^.

### Imaging results

**Figure 2:**
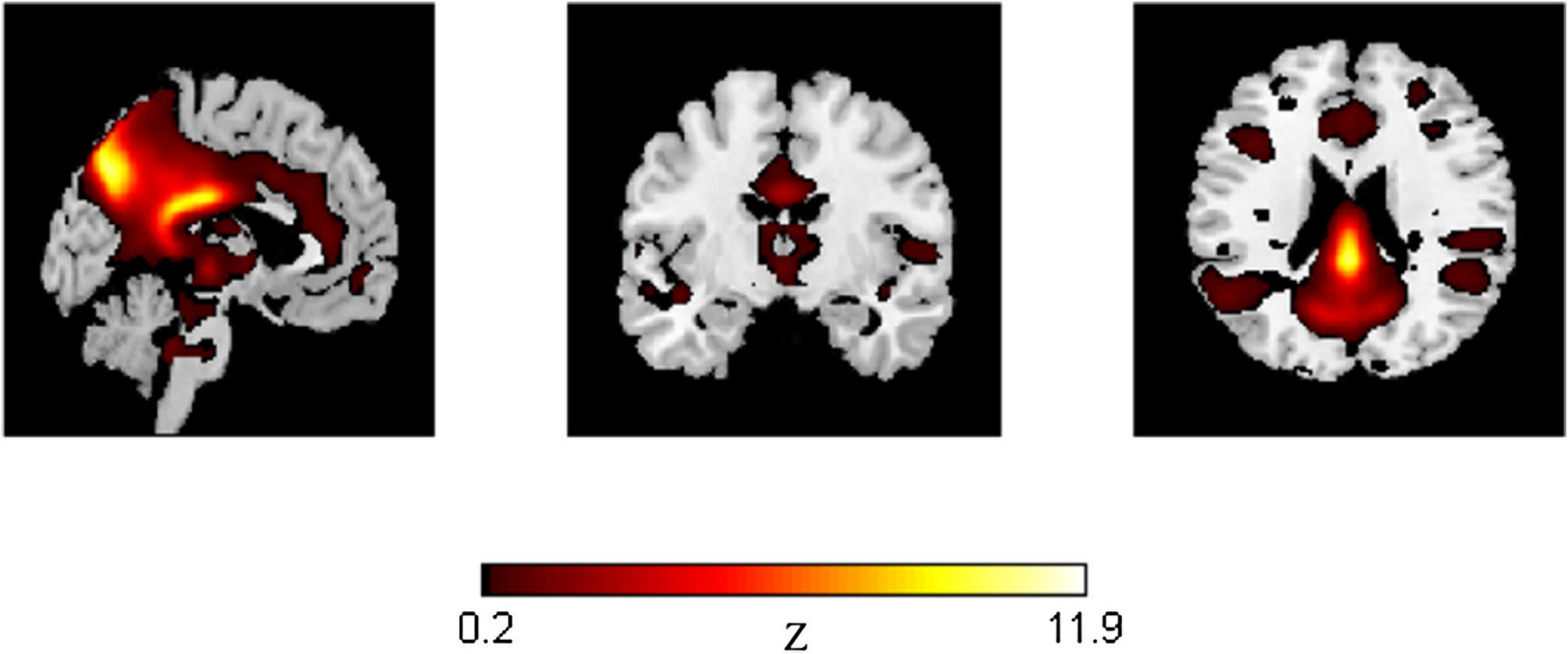
Mean group statistical map of the spatial component of the ventral DMN given by ICA analysis in GIFT toolbox.

To study group specific potential functional alterations across time on the DMN (Fig. 2), an ICA was performed.

#### Dual- and single-task training group vs control group over time in the ventral DMN

**Figure 3:**
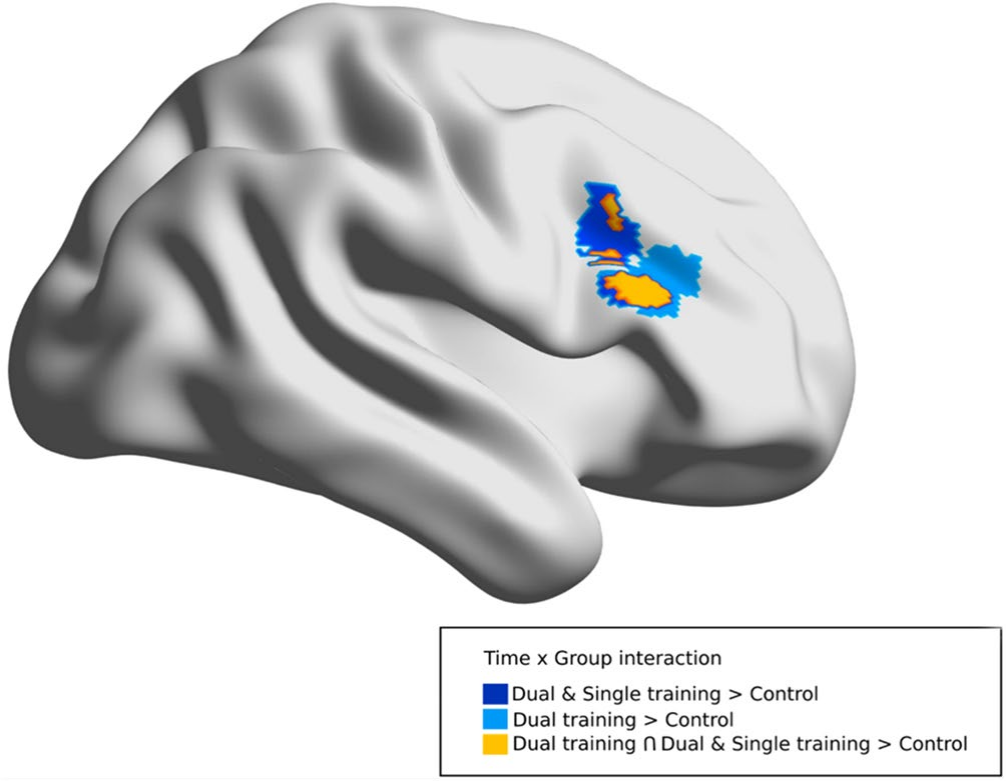
Illustration of the time × group interaction within the ventral DMN. When considering the effect of training (pooling dual and single-task) compared to control group over time, there was an increase in the connectivity over time in the right inferior frontal gyrus (rIFG; in dark blue). To analyze the effect of specific training conditions, a post hoc analysis compared dual-task training versus control group over time and single-training versus control group over time. For dual-task training versus control group, there was an increase in the connectivity in the rIFG (in light blue) overlapping with the results for the pooled training group versus control group (in orange).

When running the time × group interaction contrast for dual-task training versus single-task training as well as for dual-and-single-task training versus the control group, we found a significant effect for the contrast testing of training gains in the pooled data of the dual- and single-task training group as compared to the control group over time in right inferior frontal gyrus (rIFG), spanning opercularis and triangularis (52, 16, 36, Table 1, Fig. 3 in dark blue) in the ventral DMN.

**Table 1:** Significant group x time interaction in functional connectivity of resting state networks.

Network	Labels	MNI coordinates	T	cluster size (in voxels)	P (cluster level FWE)
**Dual- and single-task training** > **no training**					
Ventral DMN	Right inferior frontal gyrus, opercularis/triangularis	52, 16, 36	4.41	123	0.003 (cluster level FWE)
**Dual-task training** >** no training**					
Ventral DMN	Right inferior frontal gyrus, triangularis/opercularis	58, 24, 26	4.11	94	0.015 (cluster level FWE)
**Dual-task training** < **no training**					
Ventral DMN	Left superior parietal	− 26, − 56, 58	4.54	97	0.012 (cluster level FWE)

**Figure 4:**
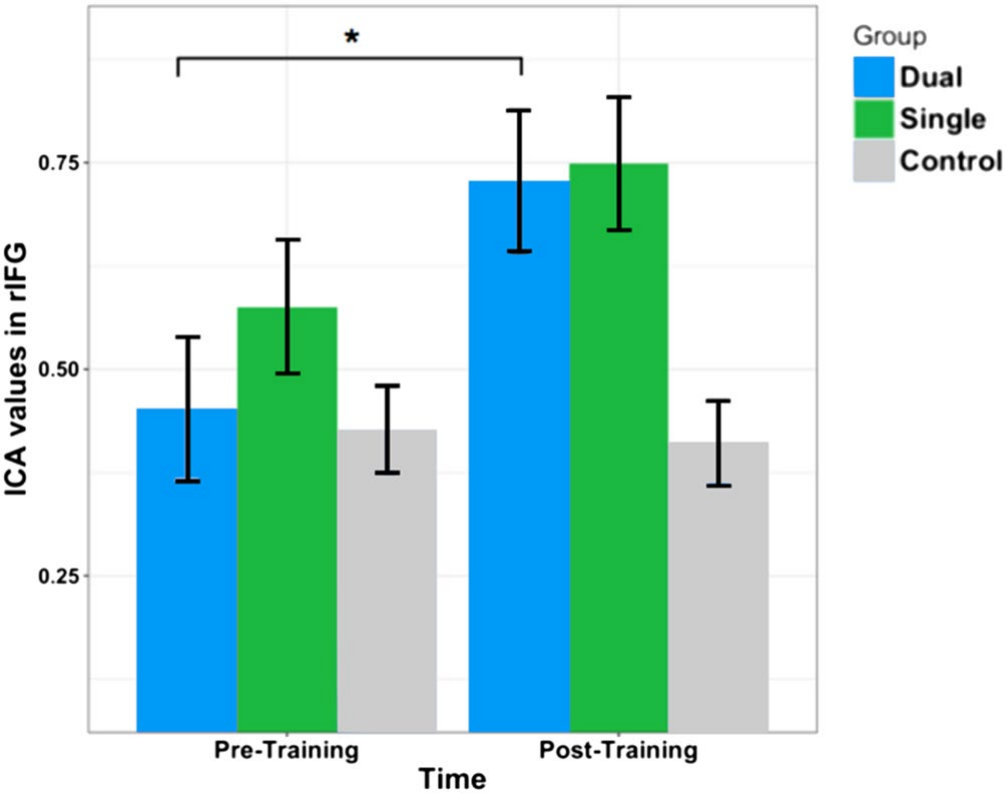
Bar plot illustrating the time × group interaction in the rIFG cluster depicted in dark blue in Fig. 3, for each group separately: dual training group (blue), single training group (green) and control group (gray).

An exploratory analysis in this cluster (Fig. 3, in dark blue) was performed in order to investigate whether the interaction effect was potentially due to a specific type of training. This analysis revealed that the interaction effect is driven by a pronounced and significant increase for the dual-task training group (*t*(17) =  − 2.23, *p* = 0.039) (Fig. 4, in blue), whereas the change in the single-task group over time was not significant (*t*(17) =  − 1.51, *p* = 0.149) (Fig. 4, in green), as the control group that remained unchanged over time (*t*(14) = 0.22, *p* = 0.831) (Fig. 4, in gray) in terms of functional connectivity of the rIFG.

#### Correlation between functional connectivity in the DMN and task performance

In order to relate the change in functional connectivity in rIFG within the ventral DMN to performance gains we computed a correlation between the gain (post–pre training) in ICA values from rIFG with task performance gain (post–pre training) in the dual-task working memory updating. When pooling both training groups (dual-task and single-task training group) we found an almost significant correlation (*r*(32) = 0.331, *p* = 0.056) controlling for age and sex. To follow up on this effect we conducted an exploratory analysis separately for each group: single-task: *r*(14) = 0.162, *p* = 0.549, dual-task: *r*(14) = 0.503, *p* = 0.047 and control: *r*(11) =  − 0.359, *p* = 0.229.

Although the positive association between performance gain and connectivity gain in the dual-task group would not survive Bonferroni correction, we see it as further evidence pointing at the fact that it is actually the dual-task training condition for which the rIFG functional connectivity increase within the ventral DMN is relevant.

#### Post hoc analysis dual-task training versus controls and single-task training versus controls

To back this up further in terms of connectivity in the ventral DMN, we performed a post hoc analysis exploring separately the contrast dual-task training versus controls and single-task training versus controls. With a threshold of *p* < 0.05 (FWE correction for multiple comparison), we found a significant group ×time interaction for the contrast dual-task training vs. control group in the rIFG (58, 24, 26, Fig. 3 in light blue) overlapping with the results of the initial analysis in this network (Fig. 3, in orange, both clusters are within the rIFG and the voxel overlap is 51% (48 voxels out of 94). In the reverse contrast (control > dual-task training, *p* < 0.05 FWE) we found a significant effect in left superior parietal cortex (− 26, − 56, 58–k = 97) indicating a decreased connectivity of the dual-task training group over time. We found no significant effects in the contrast single-task training versus control group.

In order to test whether the group × time interaction in the connectivity within the ventral DMN was not due to differences already present in the pretest sessions, we contrasted the pre-test of single-task and the pre-test of the dual-task. We did not find statistically significant differences between the pre-tests (*p* > 0.05 FWE on cluster level).

#### More resting state networks

In studies using ICA, usually only the DMN is analyzed. Nevertheless, ICA can also separate the signal of more resting state networks. These signals are important because they are being processed by the brain during rest as well. Therefore, we performed an exploratory analysis considering the spatial maps of other networks revealed by ICA: basal ganglia, sensorimotor, auditory, visuospatial, anterior salience and precuneus. None of these networks was affected by the WM training.

## Discussion

We studied changes in resting state functional connectivity in the ventral DMN in participants subjected to different WM training interventions. Following training with the cognitively demanding dual n-back task, participants showed increased functional connectivity of the ventral DMN in the rIFG. Interestingly, the prefrontal cortex is one of the key regions in the DMN. This finding was driven by a significant increase in the dual n-back training group, whereas the single n-back training group did not show significant changes between pre- and post-assessments. The dual n-back training group additionally showed decreased functional connectivity at rest in the left superior parietal cortex.

These results show that WM training with additional load on the cognitive system not only alters the task-related neuronal activity as we reported in a previous study^6^ but also neural activation in the resting-state networks of the brain.

The alterations in resting functional connectivity following cognitive training shown in our results are in accordance with previous studies. However, our results also extend the findings by showing differential effects in the resting-state functional connectivity changes according to training complexity. The changes in the resting-state characteristics of the ventral DMN were specific to the dual n-back training group. The dual n-back is a training task that required the engagement of several different cognitive functions, differently from the pre- to post-assessment changes in the single n-back training group that did not significantly exceed the effects in the no-training control group.

It is also noteworthy, that in the current study the effects were observed already after 16 days of WM training. In a previous study that reported resting-state activation changes following WM training, the participants trained for 27 days^18^. Therefore, functional reorganization of the brain’s default state can occur already after relatively short interventions, given that the intervention is sufficiently demanding for the cognitive system, such as the dual n-back task.

Taking these findings together, we showed that the training-related changes in task-unrelated spontaneous neural activations are associated with the complexity of the training paradigm. These results imply that only a cognitively demanding task that produces strong enough neuronal activation can produce such plasticity in the default mode of the brain networks that are associated with the cognitive demands of the training task.

Interestingly, plasticity in the brain’s resting functional efficiency in higher-level cognitive networks has previously been shown after physical exercise^23^. From the current data, it cannot be excluded that similar effects would be observed after single n-back training if the training period was longer. This issue should be investigated further, especially in light of the results of a meta-analysis by Soveri et al.^29^, which compared studies that investigated the effects of either dual n-back training or single n-back training (i.e., effects between studies); there were no differences found between the two training types.

We would like to note that we found a trend for a correlation between the resting-state functional connectivity of the ventral DMN in right IFG and improved behavioral performance. Although this finding should be interpreted with caution, it is consistent with previous studies that have shown resting-state activations to be correlated with behavioral measures. For example, Reineberg et al.^11^ showed that resting-state brain activity is associated with different executive functions. In this regard the current observation of a trend for a significant correlation between the connectivity at rest and behavioral performance in an untrained WM transfer task might be informative. In particular, this finding could imply that increased connectivity provided benefits in WM processes that were not purely tied to the trained task but rather to WM processes on a more general level.

Therefore, the effects of cognitive training seem not only to be reflected in the fluctuations of neural functions during a task, but also in the functional connectivity of the brain’s intrinsic default state. The resting-state functional connectivity changes possibly reflect changes in the brain’s architecture and metabolism following training, in our case, more specifically, decrease in the left superior parietal cortex and increase in the rIFG.

The rIFG has been associated in previous studies with inhibition and attentional control processes^30^, and the parietal cortex has been associated with WM processes^31^. All of these functions are extensively required in the training task of the current study and, moreover, the pattern of the activation changes is likely to reflect the functional reorganization of these processes that also lead to behavioral performance changes.

When it comes to connectivity, increasing connectivity in prefrontal regions has been suggested to reflect a dominant contribution of an anterior executive DMN hub, the role of which is strengthened as a result of the current WM training and correlates with WM efficiency^32^. This is accompanied by the observation of a relatively stronger connectivity in superior parietal regions in the control group if compared to the connectivity after WM training^9^) Whether this is indicative for training-related differences in local and executive WM processes and their role in the current dual n-back task^3,5,6^ is speculative and must be subject to further studies.

The current resting-state activation changes extend previously reported task-related functional changes and microstructural changes in white matter pathways following dual n-back training^6,9^. They also provide further evidence towards the effects of cognitive training on neural plasticity. Despite the fact that the functional role of spontaneous neural activations during rest is still unclear, several possible explanations have been offered. Spontaneous neural activations have been suggested to reflect memory consolidation processes^33^, the coordination and organization of neuronal activity between regions that have commonly been engaged during a task^34,35^, as well as the prediction of expected synchronized activations^36^. These mutually non-exclusive hypotheses all offer intriguing speculations on the underlying mechanisms of cognitive interventions, as seen in the activation changes in the resting brain. An improved communication between the parts of the network during rest seems to be an intriguing possibility for causality and also for consequences of the training-related changes in the neural substrate after intensive working memory training.

Summarizing, we conclude that WM training benefits stem from various changes in the underlying neural substrate, with the current results providing further evidence on how training interventions trigger neural changes that can even be observed in the default state of the brain.

## Supplementary information

Supplementary Information.
