# Supplementary material for: Dual n-back training improves functional connectivity of the right inferior frontal gyrus at rest
Source: Sci Rep. 2020 Nov 23;10:20379. doi: 10.1038/s41598-020-77310-9 (PMC7683712; doi:10.1038/s41598-020-77310-9)
Supplement: Supplementary file 1 — Supplementary Information. [file 41598_2020_77310_MOESM1_ESM.pdf]

Dual n-back training improves functional connectivity of the right inferior frontal gyrus at rest

Tiina Salminen[1*], Caroline Garcia Forlim[2*], Torsten Schubert[1,4], & Simone Kühn[2,3]

Supplementary material:

Table S1. Significant group x time interaction using partitioned error model showing increase in functional connectivity in the DMN for the group that underwent n-back training as compared to no training.

Network	Labels	MNI coordinates	T	cluster size (in voxels)	p
Dual- and single-task training > no training					
Ventral DMN	right inferior frontal gyrus, opercularis/ triangularis	48, 16, 30	4.41	130	0.002 (cluster level FWE)
Dual-task training > no training					
Ventral DMN	right inferior frontal gyrus, triangularis/ opercularis	48, 26, 18	5.53	70	0.034 (cluster level FWE)
